# CNOT3 suppression promotes necroptosis by stabilizing mRNAs for cell death-inducing proteins

**DOI:** 10.1038/srep14779

**Published:** 2015-10-06

**Authors:** Toru Suzuki, Chisato Kikuguchi, Sahil Sharma, Toshio Sasaki, Miho Tokumasu, Shungo Adachi, Tohru Natsume, Yumi Kanegae, Tadashi Yamamoto

**Affiliations:** 1 Cell Signal Unit, Okinawa Institute of Science and Technology, 1919-1 Onna-son, Okinawa 904-0495, Japan; 2 Imaging and Instrumental Analysis Section, Okinawa Institute of Science and Technology Graduate University, 1919-1 Onna-son, Okinawa 904-0495, Japan; 3 Molecular Profiling Research Center for Drug Discovery, National Institute of Advanced Industrial Science and Technology, Tokyo 135-0064, Japan; 4 Laboratory of Molecular Genetics, Institute of Medical Science, University of Tokyo, Tokyo 108-8639, Japan

## Abstract

The CCR4-NOT complex is conserved in eukaryotes and is involved in mRNA metabolism, though its molecular physiological roles remain to be established. We show here that CNOT3-depleted mouse embryonic fibroblasts (MEFs) undergo cell death. Levels of other complex subunits are decreased in CNOT3-depleted MEFs. The death phenotype is rescued by introduction of wild-type (WT), but not mutated CNOT3, and is not suppressed by the pan-caspase inhibitor, zVAD-fluoromethylketone. Gene expression profiling reveals that mRNAs encoding cell death-related proteins, including receptor-interacting protein kinase 1 (RIPK1) and RIPK3, are stabilized in CNOT3-depleted MEFs. Some of these mRNAs bind to CNOT3, and in the absence of CNOT3 their poly(A) tails are elongated. Inhibition of RIPK1-RIPK3 signaling by a short-hairpin RNA or a necroptosis inhibitor, necrostatin-1, confers viability upon CNOT3-depleted MEFs. Therefore, we conclude that CNOT3 targets specific mRNAs to prevent cells from being disposed to necroptotic death.

Transcriptional and post-transcriptional regulation of gene expression is important for executing biological processes, and their dysregulation causes various physiological disorders, such as cancer and diabetes. Post-transcriptional regulation largely contributes to determining the quality and quantity of translatable mRNAs. Typically, a deadenylase that shortens poly(A) tail length diminishes gene expression by reducing the stability and limiting the translation of mRNAs^1^.

The large, multimeric CCR4–NOT complex is a major deadenylase that is conserved from yeast to humans^2^,^3^. In mammals, this complex consists of four catalytic subunits and at least six non-catalytic subunits. The former include exonuclease-endonuclease-phosphatase (EEP) family proteins (CNOT6 and CNOT6L) and DEDD (Asp-Glu-Asp-Asp) family proteins (CNOT7 and CNOT8), while the latter includes CNOT1, CNOT2, CNOT3, CNOT9, CNOT10 and CNOT11. Accumulating evidence suggests that each subunit, both catalytic and non-catalytic, plays an important physiological role. In case of the catalytic subunits, the short-hairpin RNA (shRNA)-mediated depletion of CNOT6L in NIH3T3 cells induces G1 arrest^4^. Depletion of mouse CNOT7, a catalytic subunit of the CCR4-NOT complex, suppresses spermatogenesis and confers male sterility^5^,^6^. Furthermore, simultaneous suppression of CNOT6, CNOT6L, CNOT7 and CNOT8 induces apoptosis in HeLa cells^7^. The non-catalytic subunits, CNOT1, CNOT2 and CNOT3, appear to control deadenylase activity. For instance, microRNA (miRNA)-dependent deadenylation is suppressed in CNOT1-depleted *Drosophila melanogaster*^8^,^9^. CNOT2 depletion also affects the length of mRNA poly(A) tails^10^. CNOT1 or CNOT2 suppression in HeLa cells decreases deadenylase activity partly because of a structural disorganization of the complex^7^,^11^. In the case of CNOT3, a slight poly(A)-tail lengthening has been observed in yeast *not3* mutants^12^. *Drosophila* NOT3 interacts with Bicaudal-C, an RNA-binding protein that is maternally required for embryo patterning, and participates in recruitment of the deadenylase subunit to its target mRNA^13^. Because of CNOT3’s role in recruiting the deadenylase complex, CNOT3 haplodeficiency in mice affects stability of some mRNAs involved in energy metabolism and bone formation, due to poor recruitment of the CCR4-NOT complex to the mRNA 3′ untranslated regions (UTRs)^14^,^15^. Finally, CNOT3 also contributes to destabilization of *mad1* mRNA, which is important for proper mitotic progression. Consequently, the population of cells in mitotic arrest increases upon CNOT3 depletion^16^. Consistent with the idea that CNOT1, CNOT2 and CNOT3 serve as regulators, structural analyses have shown that CNOT1 functions as a scaffold subunit of the complex and that the CNOT2-CNOT3 heterodimer binds to CNOT1(refs 17,18). However, the mechanism by which CNOT2 and CNOT3 control CCR4-NOT deadenylase activity and their physiological roles have not been fully elucidated.

Necrosis has been considered as non-regulated cell death that occurs in response to overwhelming stress. Genetic analyses and the discovery of chemical inhibitors of necrosis have revealed the existence of genetically controlled necrotic pathways^19^. The best understood form of regulated necrosis is RIPK1-RIPK3-mediated, programmed necrosis (necroptosis). Various human inflammatory diseases, including ischemic injury, neurodegeneration, viral infection, and other tissue damage involve necroptosis^20^. Upon stimulation of death receptors such as tumor necrosis factor receptor 1 (TNFR1), necroptosis is promoted by activation of RIPK1 and formation of the RIPK1-RIPK3 complex^21^. Ligation of toll-like receptor (TLR) following pathogen infection also promotes necroptosis in which RIPK3, but not RIPK1, plays a primary role^19^. Caspase-8, initiator of the death receptor-induced apoptotic pathway, negatively regulates necroptosis by forming a complex with FLIP, a caspase-like molecule that lacks a catalytic cysteine^22^. Indeed, suppression of the caspase with the pan-caspase inhibitor, zVAD-fluoromethylketone (zVAD), facilitates TNFα- and TLR ligation-induced necroptosis in macrophages and microglia^23^,^24^. Several studies have shown that expression level of RIPK3 correlates with sensitivity to necroptosis^24^,^25^,^26^,^27^,^28^, implicating upregulation of RIPK3 level as one of the mechanisms by which cells are predisposed to necroptosis. However, little is known about how the expression levels of necroptosis executioners such as RIPK3 are regulated.

In this study, using primary MEFs that lack CNOT3, we provide evidence that CNOT3 maintains stable expression of other CCR4–NOT complex subunits and supports cell viability. We also show that a number of transcripts are stabilized and upregulated in the absence of CNOT3. Importantly, stabilized/upregulated mRNAs include those that encode proteins in the programmed necrosis pathway. Thus, we propose that CNOT3 is essential to the CCR4-NOT complex in controlling levels of cell death-inducing mRNAs.

## Results

### CNOT3 suppression in MEFs affects cell viability and CCR4-NOT complex formation

**Figure 1:**
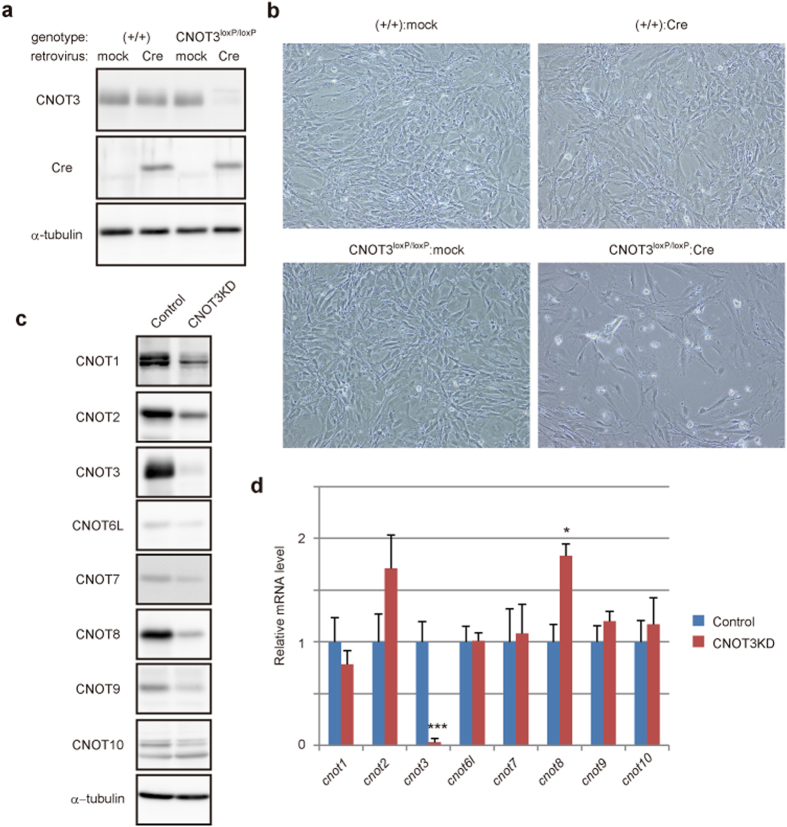
CNOT3 deficiency decreases other CCR4-NOT complex subunits and cell viability. (**a**) WT (+/+) and CNOT3^loxP/loxP^ MEFs were infected with mock- or Cre-expressing retrovirus. Cell lysates were analyzed by immunoblot. (**b**) Cell morphology 4 days after infection. Photographs are at the same magnification. Dead cells that were about to lose adhesion were observed in CNOT3^loxP/loxP^:Cre MEFs. (**c**) CNOT3^loxP/loxP^ MEFs infected with mock- (Control) or Cre-expressing retrovirus (CNOT3KD) were lysed 4 days after infection, and lysates were analyzed by immunoblot. (**d**) qPCR analysis in control or CNOT3KD MEFs 4 days after infection. *gapdh* mRNA levels were used for normalization. n = 3 for each genotype. All values represent means + sem. *P < 0.05; ***P < 0.001

*cnot3*-deficient mice die in embryo^14^, which prevented us from studying molecular physiological roles of CNOT3 in tissues and cells. To circumvent this obstacle, we used mice carrying a floxed allele of CNOT3 (CNOT3^loxP/loxP^)^14^. Primary embryonic fibroblasts were prepared from mice and were then infected with a retrovirus expressing the Cre recombinase to produce CNOT3-depleted MEFs. Immunoblot analysis revealed that CNOT3-depletion was successful (Fig. 1a). Upon CNOT3 depletion, MEFs underwent cell death, as indicated by the appearance of floating and aggregating cells (Fig. 1b). These outcomes were not observed in WT MEFs infected with mock or Cre-expressing retrovirus, or in CNOT3^loxP/loxP^ MEFs infected with mock retrovirus (Fig. 1b). Hereafter, mock virus-infected CNOT3^loxP/loxP^ MEFs are referred to as control MEFs.

As depletion of either CNOT1 or CNOT2 was shown to abrogate structural integrity of the CCR4-NOT complex^7^,^11^, we investigated whether depletion of CNOT3 affects complex integrity and found that levels of most other subunits were decreased in CNOT3-depleted MEFs (Fig. 1c). Previously, we showed that small interfering RNA (siRNA)-mediated CNOT1 suppression resulted in proteasomal degradation of other CCR4-NOT complex subunits in HeLa cells^7^. Unlike CNOT1-suppressed HeLa cells, however, treatment of CNOT3-depleted MEFs with MG132, an inhibitor of proteasomes and of calpain, had little effect on decreased levels of other subunits (Supplementary Fig. 1). While we detected a significant decrease of *cnot3* mRNA, mRNA levels of other CCR4-NOT complex subunits were not reduced in CNOT3-depleted MEFs (Fig. 1d). *cnot8* mRNA was rather significantly increased in CNOT3-depleted MEFs, but that did not result in increased CNOT8 protein (Fig. 1d). This suggested that decreased levels of these subunits in the absence of CNOT3 were due to other proteolytic activities. CNOT3 apparently maintains structural and functional integrity of the CCR4-NOT complex and supports cell viability.

### CNOT3-depleted MEFs undergo necroptosis

**Figure 2:**
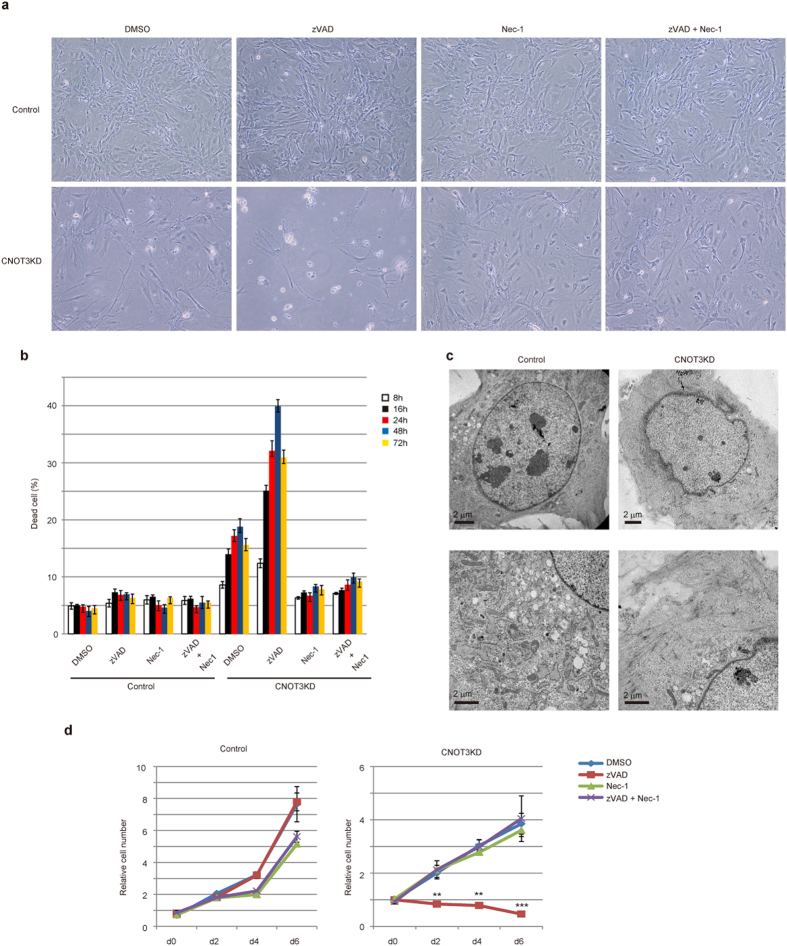
CNOT3-depleted MEFs undergo necroptosis. (**a**) Morphology of control and CNOT3-depleted MEFs (CNOT3KD) 2 days after treatment with dimethyl sulfoxide (DMSO), zVAD, Nec-1, or a combination thereof. Photographs are at the same magnification. (**b**) Cell death assessed by propidium iodide (PI) uptake using flow cytometry of treated MEFs at the indicated time points after an addition of the indicated chemicals. n = 3. (**c**) Transmission electron microscopy of control (left panels) and CNOT3KD MEFs (right panels). (**d**) Growth curves corresponding to control (left) and CNOT3KD MEFs (right) treated with indicated chemicals. Day 0 corresponds to 4 days after retrovirus infection. Each time point was determined in triplicate. All values represent means ± sem. **P < 0.01; ***P < 0.001

Because apoptosis is induced by suppression of CNOT1 or CNOT2 in HeLa cells^7^,^11^, we speculated that depletion of CNOT3 might have a similar effect in MEFs. To investigate whether cell death observed in CNOT3-depleted MEFs is caused by apoptosis, we treated cells with zVAD. While zVAD did not affect the viability of control MEFs, it increased cell death induced by CNOT3 depletion (Fig. 2a,b). zVAD can trigger or enhance necrotic cell death by suppressing the Caspase-8-mediated anti-necrotic pathway^23^,^29^,^30^,^31^. We then treated cells with Necrostatin-1 (Nec-1), which inhibits necroptosis^32^. Viability of CNOT3-depleted MEFs improved in the presence of Nec-1 (Fig. 2a,b, compare DMSO- and Nec-1-treated CNOT3KD). Additionally, Nec-1 efficiently inhibited zVAD-promoted death of CNOT3-depleted MEFs (Fig. 2a,b, compare zVAD- and zVAD+Nec1-treated CNOT3KD). These results indicate that CNOT3-depleted MEFs underwent necroptosis. Consistent with this finding, electron microscopy revealed that CNOT3-depleted MEFs manifested several necrotic features, such as loss of organelles and subcellular structures and disruption of the plasma membrane (Fig. 2c). Furthermore, neither caspase-8 nor caspase-3, which participate in apoptosis, was significantly activated in CNOT3-depleted MEFs (Supplementary Fig. 2). On day 3 after zVAD treatment that suppresses the anti-necroptotic pathway, surviving cell populations increased (Fig. 2b). We speculate that those cells may have escaped Cre-mediated deletion of the *cnot3* gene. Indeed, the surviving cells expressed CNOT3 at levels comparable to those in control MEFs (Supplementary Fig.3).

When treated with Nec-1 or with zVAD and Nec-1 together, growth rates of CNOT3-depleted MEFs failed to recover to the rate of control MEFs (Fig. 2d). This implies that cell proliferation is also suppressed by an additional mechanism upon CNOT3 depletion.

### Importance of the CNOT3 C-terminal region for cell viability

**Figure 3:**
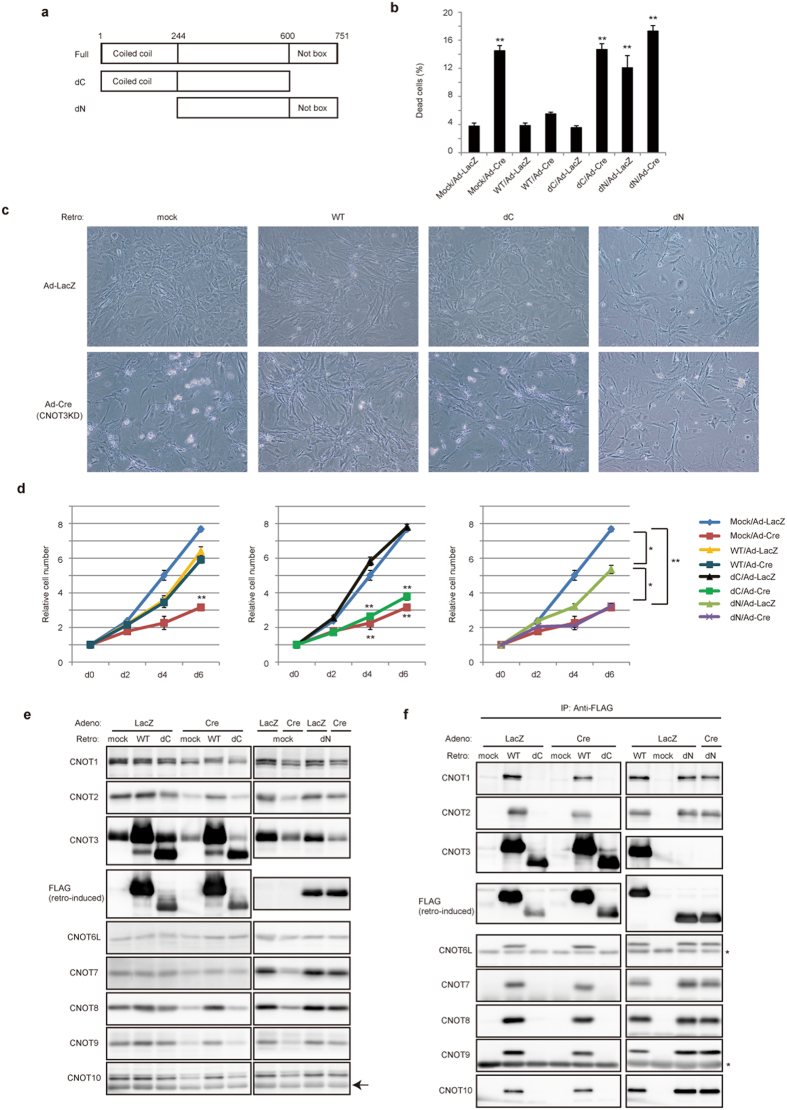
Activity of the CCR4-NOT complex depends upon a complete CNOT3 C-terminus and CNOT3 function requires an intact N-terminus. (**a**) Schematic diagram of CNOT3. CNOT3 consists of an N-terminal coiled-coil domain, a linker region and a Not box region that shares homology with CNOT2. Numbers above the protein outline represent amino acid positions at domain boundaries. (**b**) Cell death assessed by PI uptake using flow cytometry of CNOT3^loxP/loxP^ MEFs transduced with retroviruses (CNOT3 constructs)/adenoviruses (LacZ or Cre). n = 3. (**c**) Cell morphology 4 days after adenovirus infection. (**d**) Growth curves corresponding to CNOT3^loxP/loxP^ MEFs transduced with retroviruses (CNOT3 constructs)/adenoviruses (LacZ or Cre). Day 0 corresponds to 2 days after adenovirus infection. Each time point was determined in triplicate. Growth curves of control cells, Mock/LacZ (blue line) and Mock/Cre (red line), are blotted to every graph for comparison. All values represent means ± sem.*P < 0.05; **P < 0.01. (**e**) Lysates were prepared from cells infected with retroviruses (CNOT3 constructs) and adenoviruses (LacZ or Cre) and analyzed by immunoblot. The arrow indicates a nonspecific signal. (**f**) Lysates prepared in (**e**) were immunoprecipitated with anti-FLAG antibody and immunoprecipitates were analyzed by immunoblot. *IgG heavy and light chains.

The C-terminal region of CNOT3, known as the Not box, is required for its interaction with both CNOT1 and CNOT2 (ref. 33). We investigated whether the Not box is necessary for cell viability and for maintaining complex integrity. To do so, we introduced either FLAG epitope-tagged WT CNOT3 or a CNOT3 mutant lacking the 145 C-terminal amino acids (the Not box) (mutant dC, Fig. 3a) into CNOT3-depleted MEFs using a recombinant retrovirus. In this set of experiments, we used a recombinant adenovirus expressing Cre to delete the *cnot3* gene^34^. When WT CNOT3 was reintroduced into CNOT3-depleted MEFs, cell viability was comparable to that of control MEFs (Fig. 3b,c). Reintroduction of WT CNOT3 also rescued the growth capacity of CNOT3-depleted cells (Fig. 3d, left graph). In contrast, expression of CNOT3dC did not rescue these defects (Fig. 3b–d). We also found that introduction of WT CNOT3, but not CNOT3dC, in CNOT3-depleted MEFs rescued expression of all CCR4-NOT complex subunits (Fig. 3e, left panels). Importantly, immunoblot analysis of anti-FLAG immunoprecipitates from CNOT3-depleted cell lysates revealed that CNOT3 was associated with all other subunits when WT CNOT3 was reintroduced, suggesting the formation of complete CCR4-NOT complexes (Fig. 3f, left panels). In contrast, complex formation did not appear to occur in CNOT3dC-introduced cells (Fig. 3f, left panels). These data suggest that the C-terminal region of CNOT3 is necessary for formation of a complete CCR4-NOT complex and for maintaining the expression of other subunits. In summary, the C-terminal region of CNOT3 is indispensable for CCR4-NOT complex integrity and cell viability.

### Role of the N-terminal coiled-coil region of CNOT3 in cell viability

Next, we introduced a CNOT3 mutant lacking the N-terminal 245-amino acid coiled-coil region (mutant dN: Fig. 3a) into CNOT3-depleted MEFs. As with CNOT3dC expression, necroptosis of CNOT3-depleted MEFs was not suppressed by expression of CNOT3dN (Fig. 3b,c). Expression of COT3dN also failed to rescue CNOT3-depleted MEF proliferation (Fig. 3d, right graph). Intriguingly, introduction of CNOT3dN itself induced cell death and partially suppressed cell proliferation, even in control MEFs, which was most likely due to the dominant-negative effect of CNOT3N (Fig. 3b–d). Therefore, we tentatively conclude that the coiled-coil region of CNOT3 is necessary for cell viability. We also found that expression levels of all subunits, except CNOT3, was rescued by CNOT3dN (Fig. 3e, right panels). Additionally, all CCR4-NOT complex subunits co-immunoprecipitated with CNOT3dN (Fig. 3f, right panels), indicating that the N-terminal coiled-coil region in CNOT3 is dispensable for stable expression of the other subunits and their assembly.

### Suppression of CNOT3 stabilizes a set of transcripts

**Figure 4:**
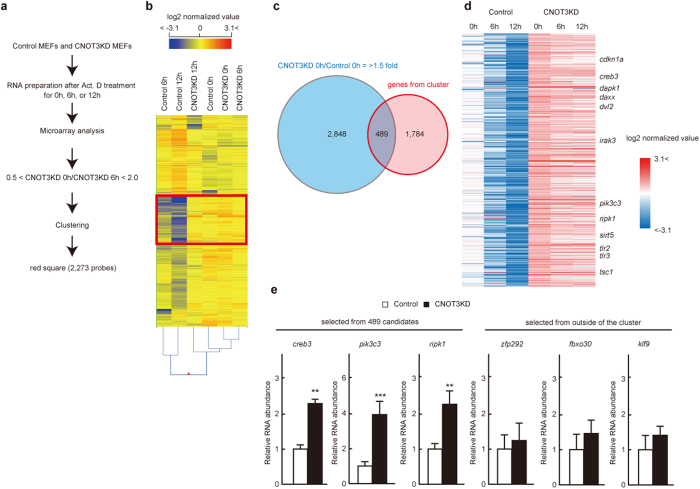
Microarray analysis of RNA following transcriptional inhibition identifies CNOT3-regulated mRNAs. (**a**) Procedure for selection of genes, the stability of which is affected by CNOT3 suppression. (**b**) Heat map after a hierarchical clustering. A red square indicates a cluster in which transcripts are largely decreased in controls, but not CNOT3KD MEFs after Act. D treatment. (**c**) Venn diagram illustrating the overlap between the gene selected from (**b**) (circled red) and genes up-regulated >1.5 fold in CNOT3KD MEFs compared with control MEFs (circled blue). (**d**) Heat map showing relative expression levels of the overlapped 489 genes in (**c**). (**e**) qPCR analysis of mRNAs in control or CNOT3KD MEFs. *gapdh* mRNA levels were used for normalization. n = 3 for each genotype. All values represent means + sem. **P < 0.01; ***P < 0.001

Because CNOT3 is involved in regulation of CCR4-NOT deadenylase activity and thereby controls mRNA levels^14^, we compared gene expression profiles between control and CNOT3-depleted MEFs using Affymetrix microarray technology to elucidate the molecular basis of necroptosis in CNOT3-depleted MEFs. Total RNA samples were prepared from MEFs treated with the transcriptional inhibitor, actinomycin D (Act. D), for 6 and 12 h (Fig. 4a). Hierarchical clustering of gene sets revealed that one cluster of 2,273 transcript probe sets was highly stabilized in CNOT3-depleted MEFs compared with control MEFs (Fig. 4b; Supplementary Table 1). We hypothesized that a significant population, though perhaps not all of these mRNAs, are CNOT3 targets. We then searched for mRNAs that were more highly expressed in CNOT3-depleted MEFs than control MEFs without Act. D treatment. Among the 2,273 probe sets, 489 corresponded to genes that were upregulated more than 1.5-fold in CNOT3-depleted MEFs compared with control MEFs (Fig. 4c; Supplementary Table 2). The upregulated mRNAs encoded proteins involved in regulation of cytoplasm organization, autophagy, cell death, and cell proliferation (Supplementary Table 3). Importantly, necroptosis-related genes, such as *ripk1*, *irak3*, *tlr2* and *tlr3* were included among the upregulated genes (Fig. 4d; Supplementary Table 3). By examining levels of these mRNAs using quantitative real-time polymerase chain reaction (qPCR), we found that *creb3*, *pik3c3*, *ripk1*, *dvl2*, *sirt5* and *cdkn1a* mRNA levels were significantly elevated in CNOT3-depleted MEFs (Fig. 4e; Supplementary Fig. 4A). We anticipated that the upregulated mRNAs might also include an important necroptosis-related gene, *ripk3* mRNA. While *ripk3* mRNA was recognized by the microarray analysis, it was present at such low levels in WT MEFs that it was impossible to determine whether there had been a change in expression level. Therefore, we directly measured *ripk3* mRNA by qPCR to ascertain its higher expression level in CNOT3-depleted MEFs (Supplementary Fig. 4A). Consistent with this, both RIPK1 and RIPK3 proteins increased following CNOT3 depletion (Supplementary Fig. 4B). Note that transcripts, such as *fbxo30*, *klf9* or *zfp292*, that have short half-lives both in the presence and absence of CNOT3 in microarray results, were almost the same in control and CNOT3-depleted MEFs (Fig. 4e).

### mRNAs stabilized in the absence of CNOT3 had longer poly(A) tails

**Figure 5:**
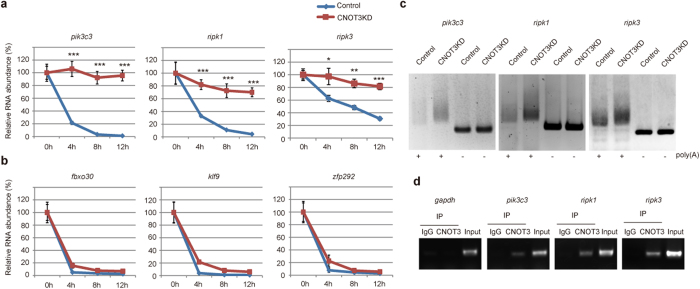
Reduced poly(A) tail shortening in CNOT3-depleted MEFs stabilizes target mRNAs. (**a**,**b**) Control and CNOT3KD MEFs were treated with Act. D. Relative mRNA levels were determined by qPCR at 4 h time intervals after Act. D treatment and normalized to the *gapdh* mRNA level. mRNA level without Act. D treatment (0 h) was set to 100%. n = 3 for each genotype. All values represent means ± sem. *P < 0.05; **P < 0.01; ***P < 0.001 (**c**) Comparison of poly(A) tail length of mRNAs between control and CNOT3KD MEFs. Total cellular RNA was subjected to PCR-based analysis (see Methods). PCR products without poly(A) regions (−) were also loaded. (**d**) Lysates from MEFs were immunoprecipitated with control Ig or anti-CNOT3 antibodies. Immunoprecipitates were analyzed by RT-PCR using primers for 3′UTR in the indicated genes.

We analyzed the time course of mRNA expression level following Act. D treatment and found that half-lives of *pik3c3*, *ripk1*, *ripk3*, *creb3*, *dvl2*, *sirt5* and *cdkn1a* mRNAs were prolonged in CNOT3-depleted MEFs (Fig. 5a; Supplementary Fig. 5A). In contrast, half-lives of *fbxo30*, *klf9* and *zfp292* mRNAs were similar between control and CNOT3-depleted MEFs (Fig. 5b). To verify that stabilization of mRNAs in the absence of CNOT3 is a consequence of poor deadenylation by the CCR4-NOT complex, we analyzed poly(A) tail lengths of *pik3c3*, *ripk1* and *ripk3* mRNAs. Their poly(A) tails were significantly longer in CNOT3-depleted MEFs than control MEFs (Fig. 5c). Furthermore, reverse transcription (RT)-PCR analysis of RNAs that immunoprecipitated with anti-CNOT3 antibody showed that CNOT3 physically interacts with *pik3c3*, *ripk1*, *ripk3*, *creb3*, *dvl2*, *sirt5* and *cdkn1a* mRNAs (Fig. 5d; Supplementary Fig. 5B). Previous reports showed that the CCR4-NOT complex utilizes the miRNA/Argonaute (Ago) complex and/or an AU-rich element binding protein, such as Zfp36L1, to recognize target mRNAs^35^,^36^. We found that Zfp36L1 bound to the 3′ UTR of *ripk1* and *creb3* mRNAs that possess AU-rich elements *in vitro* (Supplementary Fig. 6A,B). Furthermore, Ago2 bound to the 3′ UTR of *pik3c3*, *ripk1* and *creb3*mRNA (Supplementary Fig. 6B). These findings suggest that CNOT3 recognizes 3′ UTR sequences of *pik3c3*, *ripk1* and *creb3* mRNAs in a manner that is dependent on the miRNA/Ago complex and/or Zfp36L1.

### Relevance of CNOT3 N- and C-terminal domains in CNOT3-dependent mRNA decay

**Figure 6:**
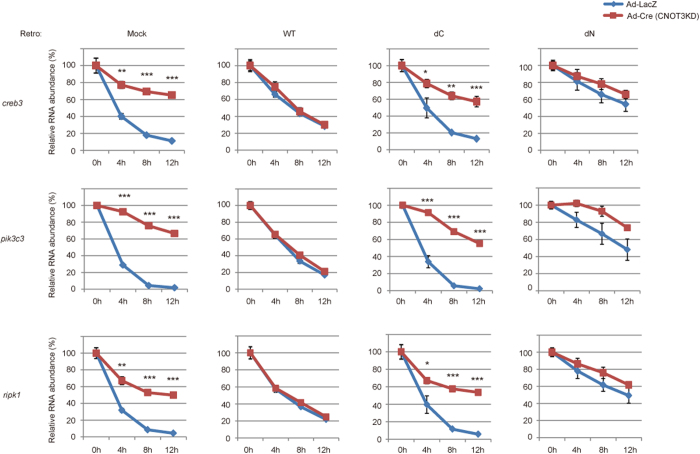
Both an N-terminal and C-terminal region are required for CNOT3-dependent mRNA decay. Decay curves of CNOT3 targets in CNOT3^loxP/loxP^ MEFs transduced with retroviruses (mock or CNOT3 constructs) and adenoviruses (Ad-LacZ or Ad-Cre) determined as in Fig. 4a. n = 3. WT CNOT3 expression in CNOT3KD MEFs (red lines in left middle graphs), but not CNOTdC and CNOTdN expression (red lines in right middle and rightmost graphs) restored MEF half-lives to control levels (blue lines in leftmost graph. CNOT3dN expression in control MEFs stabilized mRNAs (compare blue lines in between leftmost and rightmost graphs). All values represent means ± sem. *P < 0.05; **P < 0.01; ***P < 0.001

Next, we reintroduced WT CNOT3 into CNOT3-depleted MEFs. The elongated half-lives of *creb3*, *pik3c3* and *ripk1* mRNAs were shortened and were comparable to those of control MEFs (Fig. 6, left middle). In contrast, expression of CNOT3dC or CNOT3dN did not restore the half-lives of these mRNAs to control levels (Fig. 6, right middle and rightmost). Moreover, expression of CNOT3dN in control MEFs resulted in a significant stabilization of transcripts (Fig. 6, compare blue lines between Mock and CNOT3dN expressing MEFs). We noted that overexpression of WT CNOT3 transgene in MEFs resulted in stabilization of target mRNAs, albeit slightly, compared to mock retrovirus-infected MEFs (Fig. 6). By performing gel filtration analysis of lysates from control and WT-CNOT3-transduced cells, we found that overexpressed WT CNOT3 formed a smaller complex (~600 kDa) in addition to intact CCR4-NOT complex (Supplementary Fig. 7A). The smaller complex was purified by immunoprecipitation using anti-Flag antibody and the proteins in the complex were subjected to SDS polyacrylamide gel electrophoresis. Silver staining of the gel revealed that CNOT3 was predominantly present, suggesting that the ~600 kDa complex represent a CNOT3 oligomers (Supplementary Fig. 7B). These data suggested that the smaller complex is a CNOT3 oligomer that would interfere with function of the CCR4-NOT complex. Collectively, the effect of CNOT3 (WT and mutants) on cell viability is clearly correlated with its ability to degrade target mRNA levels.

### The RIPK-mediated pathway is involved in necroptosis following CNOT3 depletion

**Figure 7:**
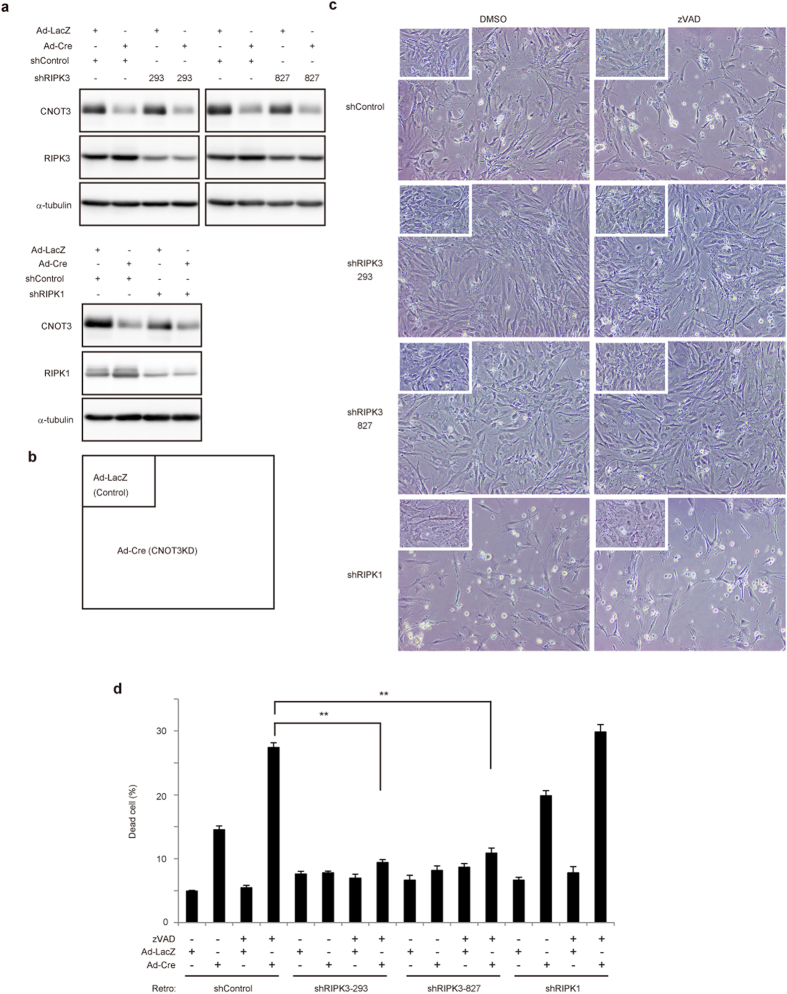
CNOT3-depleted MEFs undergo necrosis via the RIPK3-dependent death pathway. (**a**) Lysates were prepared from MEFs which were infected with retrovirus expressing shRNAs (shControl, shRIPK3-293, shRIPK3- 827 or shRIPK1) and adenovirus (LacZ or Cre) were analyzed by immunoblot. (**b**) Schematic diagram of positions of photographs. (**c**) Morphology of CNOT3KD MEFs and control MEFs (Insets) infected with retrovirus-expressing shRNAs which are treated with DMSO or zVAD for 24 h. (**d**) Cell death assessed by PI uptake via flow cytometry of MEFs transduced with retrovirus (shRNA constructs) and adenovirus (LacZ or Cre) 24 h after an addition of DMSO (−) or zVAD (+). n = 3. All values represent means ± sem. **P < 0.01

The upregulation/stabilization of *ripk1*, *ripk3* and mRNAs for other necrosis-related proteins in CNOT3-depleted MEFs appeared to be the cause of necroptosis (Figs 4d,e and 5a; Supplementary Fig.3). While RIPK1 associates with and activates RIPK3 to promote necroptosis upon stimulation of TNFR1 (ref. 21), RIPK1 inhibits necroptosis through the action of Caspase-8-FLIP complex when RIPK3 is activated independently, such as by TLR ligation or interferon (IFN) stimulation^37^. Because both mRNAs encoding proteins involved in RIPK1-dependent and RIPK1-independent necroptosis were stabilized in CNOT3-depleted MEFs (Fig. 4d; Supplementary Table 3), we first investigated the effect of expressing shRNA specific for RIPK3 (shRIPK3) on necroptosis in CNOT3-depleted MEFs. We used two different shRNAs that target different sequence segments of RIPK3. Expression of shRIPK3-293 results in strong silencing of RIPK3 (48% reduction), while shRIPK3-827 is less effective in this regard, reducing RIPK3 expression to near to normal levels (16% reduction) in CNOTKD MEFs (Fig. 7a). In the presence of CNOT3, the viability of MEFs in which shRIPK3 was introduced, was similar to that of control shRNA-introduced MEFs (Fig. 7c, insets: compare left top with middle two panels, 7d). Upon CNOT3 depletion, the number of dead cells increased among control shRNA-introduced MEFs, but not in MEFs treated with shRIPK3 (Fig. 7c, compare left top with middle two panels, 7d). Furthermore, many CNOT3-depleted, shRIPK3-treated MEFs were viable in the presence of zVAD, whereas zVAD treatment of CNOT3-depleted MEFs expressing control shRNA resulted in extensive cell death (Fig. 7c, compare right top with middle two panels, 7d). Suppression of RIPK3 significantly, though not completely, restored the proliferation rate of CNOT3-depleted MEFs (Supplementary Fig. 8). The results are similar to the effect of Nec-1 on cell proliferation (Fig. 2d). Importantly, necroptosis is significantly suppressed by both shRIPK3-827 and shRIPK3-293, indicating that reduction of RIPK3 to normal levels in CNOT3KD is sufficient to protect MEFs from necroptosis. These results suggest that CNOT3 suppression-induced stabilization of *ripk3* mRNA contributes to necroptosis induction. In contrast, when we suppressed expression of RIPK1 using shRNA specific for RIPK1 (shRIPK1), death of CNOT3-depleted MEFs was promoted rather than inhibited (Fig. 7c, bottom left, 7d). This likely occurred because the anti-necroptotic function of the Caspase-8-FLIP complex could not be promoted by RIPK1 silencing. Similar effects were observed in the presence of zVAD together with shRIPK1 (Fig. 7c, bottom right, 7d).Note that Nec-1 does not inhibit necroptosis in the absence of RIPK1 and Nec-1-bound RIPK1 plays a critical role in necroptosis inhibition^37^. Consistent with this, necroptosis observed in CNOT3KD MEFs was not suppressed by Nec-1 when RIPK1 was silenced (Supplementary Fig. 9).

## Discussion

The CCR4-NOT complex regulates gene expression at various steps, such as transcription in the nucleus and mRNA decay or quality control of translated proteins in the cytoplasm^2^,^3^. Recent evidence has suggested that a subunit of the CCR4-NOT complex, CNOT3, plays critical roles in several biological events, including oogenesis, self-renewal of embryonic stem cells, heart function, energy metabolism and bone formation^13^,^14^,^15^,^38^,^39^,^40^,^41^. Dysregulation in mRNA turnover caused by CNOT3 depletion appears to be a large contributor to known defects of these processes^13^,^14^,^15^. In the present study, we provide evidence that suppression of CNOT3 in MEFs results in necroptotic cell death, and in reduced formation of the CCR4-NOT complex, induced by decreased levels of the other subunits in the complex. Importantly, CNOT3 suppression stabilizes mRNAs that control cell viability, including mRNAs for TLR2, TLR3, RIPK1 and RIPK3, which can predestine cells for necroptosis. Stabilization observed in the absence of CNOT3 was, at least in some cases, primarily caused by reduced deadenylation of mRNAs targeted by the CCR4-NOT complex.

Recent structure-function analyses of CCR4-NOT have revealed that C-terminal regions of CNOT2 and CNOT3 contain a sequence called the NOT box that is utilized for heterodimerization of the two proteins and for their interaction with CNOT1 scaffold protein^33^. Thus loss of the CNOT3 NOT box prevented CNOT1-CNOT2-CNOT3 complex formation, resulting in loss of structural integrity of the whole CCR4-NOT complex and in suppression of deadenylase activity. Reduced deadenylation of mRNA poly(A) tails could stabilize mRNAs^42^. Consistent with this, we showed that deletion of the CNOT3 C-terminal region resulted in loss of structural integrity and stabilization of a set of mRNAs. Importantly, by expressing CNOT3dC in CNOT3-depleted MEFs, proper formation of the CCR4-NOT complex is abrogated, resulting in poor MEF viability (Fig. 3). In contrast, we have shown that the N-terminal domain of CNOT3 is not important for structural integrity of the complex. Interestingly, however, the N-terminal coiled-coil region of CNOT3 played an important role in controlling cell viability, as CNOT3dN expression in WT MEFs decreased their viability and stabilized CNOT3-target mRNAs (Figs 3 and 5). This may be due to the dominant negative effect of CNOT3dN. A previous study identified point mutations within the N-terminal coiled-coil region and frame shift mutations in CNOT3 in T-cell acute lymphoblastic leukemia^43^. It is intriguing to speculate that these mutations may be involved in development of leukemia by disabling CNOT3-target mRNA recognition. In addition, the CCR4-NOT targets are slightly stabilized by overexpression of WT CNOT3 (Fig. 6), suggesting that excess CNOT3 interferes with function of CCR4-NOT complex. Structural analysis showed that the CNOT3 NOT box forms homodimers in the absence of its binding partners (CNOT1 and CNOT2) (ref. 33). Consistent with this, overexpression of WT CNOT3 forms an oligomer in addition to intact CCR4-NOT complex (Supplementary Fig. 7). Structural analysis of the N-terminal coiled-coil region and full-length CNOT3 would help elucidate the mechanism by which CNOT3 is responsible for target recognition and how CNOT3 oligomer affects the endogenous CCR4-NOT complex.

This study showed that both mRNA for RIPK1, which is involved in death receptor pathway-mediated apoptosis or necroptosis^21^, and mRNA for RIPK3, which strongly promotes necroptosis when RIPK1 is suppressed^37^, were stabilized in the absence of CNOT3. The RIPK3-dependent necroptotic pathway, but not the death receptor-dependent apoptotic pathway, largely contributed to death of CNOT3-depleted MEFs (Fig. 7). Because RIPK3-dependent cell death causes defects in embryonic development^44^,^45^, the embryonic lethality of *cnot3*-deficient mice might, at least in part, be mediated by RIPK3. Although various studies have suggested that increased RIPK3 leads cell to necroptosis in inflamed tissues and TNFα-stimulated cells^24^,^25^,^26^,^27^,^28^, how the RIPK3 level is controlled remains to be addressed. Our results provide a possible mechanism by which RIPK3 expression increases when cells are predisposed to necroptosis, and suggest that some human inflammatory diseases, related to necroptosis, are caused by deregulation of the CCR4-NOT complex.

While CNOT3 was depleted by siRNA to induce partial mitotic arrest without apparent death^16^, in the present study CNOT3 was depleted by somatic recombination. Therefore, different degrees of suppression may cause different phenotypes. It is also possible that the effect of CNOT3 depletion varies depending on the cell types involved. HeLa cells do not express any detectable RIPK3 and do not undergo necroptosis, in contrast to other cell lines, including MEFs^25^, suggesting that stabilization of *ripk3* mRNA upon CNOT3 depletion, if any, is not enough to promote necroptosis. Further studies should elucidate the tissue- or tumor cell-specific function of the CCR4-NOT complex.

In addition to cell death-related genes, mRNAs for molecules involved in cell cycle regulation (*cdkn1a* and *id2*), autophagy (*pik3c3* and *tsc1*) and cytoskeletal organization (*arhgef18* and *myo3b)*, were also stabilized in CNOT3-depleted MEFs (Supplementary Table 3). Importantly, even after suppression of necroptosis, growth of CNOT3-depleted MEFs was retarded. Furthermore, CNOT3-depleted MEFs were enlarged, suggesting that cytoskeletal disorganization had occurred, although this disorganization may be a secondary effect of cell death or growth retardation. There are reports that the autophagic response is activated concomitantly with non-apoptotic cell death^46^,^47^. Therefore, we speculate that suppression of CNOT3 in MEFs induces various biological responses, most of which are likely to be due to stabilization of relevant mRNAs.

Cell death is correlated with nutrient and energy conditions *in viv*o and *in vitro*. Both necroptosis and apoptosis are often observed inside tumors, where nutrient and oxygen supplies are low. In cultured cells, nutrient deprivation or growth factor withdrawal leads to apoptotic cell death^48^,^49^. When the apoptotic machinery is inhibited, necroptosis or autophagy occurs. In the case of autophagy, cells can maintain their bioenergetics and viability by consuming their own molecules^48^,^50^. These cells undergo necroptosis upon inhibition of autophagy^50^. Necroptosis is also triggered by a decrease in intracellular ATP level, elevation of Ca^2+^ level, or production of reactive oxygen species, caused by DNA damage or mitochondrial dysfunction. Some of these necroptotic processes are genetically controlled^19^. We propose that regulation of *ripk1* and *ripk3* mRNAs by the CCR4-NOT complex could explain how and when necroptosis occurs. Because CNOT3 levels in liver and white adipose tissues decrease upon fasting^14^, CNOT3 may determine cell fate by regulating expression levels of cell death-related genes in response to nutrient conditions or intracellular ATP levels. Moreover, because RIPK3 regulates several metabolic enzymes, such as glycogen phosphorylase, which degrades glycogen to produce energy^23^, it is intriguing to speculate that RIPK3-dependent energy metabolism regulates the CNOT3 level in a feedback loop.

## Methods

### Cell culture

MEFs were prepared as described previously^51^ and were cultured at 37 °C in Dulbecco’s modified Eagle’s medium supplemented with 10% fetal bovine serum. zVAD-fmk, MG132 (Peptide Institute) and Nec-1 (Calbiochem) were used at 40 μM, 50 μM and 10 μM, respectively.

### Antibodies and reagents

Rabbit polyclonal antibody against CNOT1 was from Proteintech. Antibodies against CNOT2, Cleaved caspase-3, Cleaved caspase-8, RIPK1 and Zfp36L1 were from Cell Signaling Technology. Anti-Cre antibody was from Novagen. Anti-RIPK3 polyclonal antibody was from Abcam. Anti-Ago2 monoclonal antibody was from Wako. Anti-FLAG antibody and anti-α-tubulin antibody were from Sigma. Mouse monoclonal antibodies against CNOT3, CNOT6L, CNOT7, CNOT8 and CNOT9 were generated by immunizing mice with each recombinant protein in cooperation with Bio Matrix Research Incorporation. Recombinant proteins were prepared as GST-fused proteins using *Escherichia coli* and the GST portion was removed with PreScission Protease (GE Healthcare). Our CNOT3 antibody detected CNOT3dC, but not CNOT3dN, indicating that the antibody recognized the N-terminal region of CNOT3.

### Virus infection

Retrovirus (mock or Cre) infection of MEFs was performed as described previously^51^. Two days after virus infection, cells were diluted following trypsinization and cultured in the presence of puromycin (1 μg/mL) for an additional 2 days to select infected cell populations. For suppression of RIPK1 or RIPK3, MEFs were infected with retrovirus expressing shRIPK1 or shRIPK3. To construct shRNA expression vector, two synthetic oligo nucleotides were annealed and inserted into pSIREN-RetroQ vector (BD Biosciences). The target sequences for *ripk1*, *ripk3-293* and *ripk3-827* are 5′-gctggagaagacagacctaga-3′, 5′-gctggagtttgtgggtaaagg-3′ and 5′-ggcttctaaagcgagtgatgt-3′, respectively. Control MEFs were infected with retrovirus expressing shControl constructs (Morita *et al*., 2007). Adenovirus infection was performed 2 days after retrovirus infection at MOI 7.5. Two days later, infected MEFs were used for growth and cell death analysis. For immunoprecipitation and total RNA preparation, MEFs were selected with puromycin (1 μg/mL) for 2 days.

### Immunoprecipitation and immunoblotting

Cells were lysed with lysis buffer (1% NP-40, 50 mM Tris–HCl [pH 7.5], 150 mM NaCl, 1 mM EDTA, 1 mM phenylmethylsulfonylfluoride, 10 mM NaF). Lysate proteins were subjected to immunoprecipitation using anti-FLAG antibody-conjugated agarose (Sigma) and subsequent immunoblot analysis as described^51^.

### Measurement of cell survival rate

Cell viability was measured by the ability of cells to exclude propidium iodide (PI). Cells were treated with 0.1% Trypsin, collected by centrifugation, washed once with phosphate-buffered saline (PBS), and resuspended in PBS containing 5 μg/mL of PI (Sigma). Levels of PI incorporation were quantified using FACS Calibur (Becton Dickinson).

### Growth curves

Twenty-five thousand cells per well were plated into 12-well plates. At the indicated times, cells were washed with PBS, fixed in 10% formalin for 15 min, and rinsed with distilled water. Cells were stained 0.1% crystal violet (Sigma) for 30 min, rinsed extensively, and dried. Cell-associated dye was extracted with 2.0 mL of 10% acetic acid. Aliquots were transferred to 96-well plates, and optical density at 570 nm was measured. Within an experiment, each point was determined in triplicate. Each growth curve was performed at least twice.

### Microarray analysis

Total RNAs were extracted with ISOGENII according to the manufacturer’s protocol (Nippon Gene). The quality of RNA was determined using the Bioanalyzer nano chip (Agilent Technologies, Inc.). cDNA and biotin-labeled cRNA were synthesized according to the manufacturer’s instructions (Affymetrix). After fragmentation of cRNA, biotin-labeled cRNA was hybridized to the GeneChip Mouse Genome 430 2.0 Array (Affymetrix). To determine the average difference for each probe set, we used MAS5 algorithm. Selected probe set IDs were converted according to the manufacturer’s instructions. To determine fold differences in gene expression and perform a hierarchical clustering, we used GeneSpring 12.6 (Agilent Technologies). To identify enriched cellular function and pick up necrosis-related genes, we used Ingenuity Pathway Analysis (Ingenuity). The complete data set reported herein has been submitted to the NCBI GEO data base (http://www.ncbi.nlm.nih.gov/geo/) and can be obtained under accession number GSE67418.

### RNA analysis

Total RNA (1 μg) was used for reverse transcription with oligo(dT)12–18 primer (Invitrogen) using the SuperScript III First-Strand Synthesis System (Invitrogen). qPCR reactions were carried out using SYBR Premix Ex Taq (Takara) and the ABI PRISM 7900HT Sequence Detection System (Applied Biosystems). *Gapdh* mRNA levels were used for normalization. Primers for PCR reactions are listed in Supplementary Table. To measure mRNA stability, cells were treated with Act. D (2.5 μg/mL), and total RNA was extracted at the indicated times and subjected to qPCR analysis. For RNA immunoprecipitation-RT-PCR analysis, proteins in cell lysates were immunoprecipitated with anti-CNOT3, or normal mouse IgG antibodies. RNAs in immunoprecipitates were purified using ISOGENII. To compare poly(A) tail length, we used the Poly(A) Tail-Length Assay Kit (Affimetrix) according to the manufacturer’s protocol.

### Transmission electron microscopy

After a wash with PBS, MEFs were fixed in 2.5% glutaraldehyde in 0.1 M sodium cacodylate buffer for 2 h. Following three washes with 0.1 M sodium cacodylate buffer, cells were further fixed with 1% osmium tetroxide 0.1 M sodium cacodylate buffer for 1.5 h. Cells were then dehydrated in an ascending alcohol series and embedded in epoxy resin. Ultrathin sections (50 nm) of MEFs were stained with 4% uranium acetate for 30 min and subsequently with Sato’s lead staining solution. Samples were examined using a JEOL JEM-1230R transmission electron microscope.

### Statistical analyses

Differences between groups were examined for statistical significance using Student’s t-test (two-tailed distribution with two-sample equal variance). We considered a P-value of < 0.05 statistically significant.

## Additional Information

**How to cite this article**: Suzuki, T. *et al*. CNOT3 suppression promotes necroptosis by stabilizing mRNAs for cell death-inducing proteins. *Sci. Rep*. **5**, 14779; doi: 10.1038/srep14779 (2015).

## Supplementary Material

Supplementary Information

Supplementary Table S1

Supplementary Table S2

Supplementary Table S3
