# Supplementary material for: CNOT3 suppression promotes necroptosis by stabilizing mRNAs for cell death-inducing proteins
Source: Sci Rep. 2015 Oct 6;5:14779. doi: 10.1038/srep14779 (PMC4594005; doi:10.1038/srep14779)

Supplementary information

CNOT3 suppression promotes necroptosis by stabilizing mRNAs for cell death-inducing proteins

Toru Suzuki[1*], Chisato Kikuguchi[1], Sahil Sharma[1], Toshio Sasaki[2], Miho Tokumasu[1], Shungo Adachi[3], Tohru Natsume[3], Yumi Kanegae[4#], and Tadashi Yamamoto[1*]

[1]Cell Signal Unit, [2]Imaging and Instrumental Analysis Section, Okinawa Institute of Science and Technology Graduate University, 1919-1 Onna-son, Okinawa 904-0495, Japan; [3]Molecular Profiling Research Center for Drug Discovery, National Institute of Advanced Industrial Science and Technology, Tokyo 135-0064, Japan; [4]Laboratory of Molecular Genetics, Institute of Medical Science, University of Tokyo, Tokyo 108-8639, Japan

#Current address: Research Center for Medical Science, Jikei University School of Medicine, 3-25-8, Nishi-shinbashi, Minato-ku, Tokyo, 195-8461, Japan

*Correspondence: toru.suzuki@oist.jp, tadashi.yamamoto@oist.jp

Supplementary methods

Vectors

Mouse CNOT3 cDNA was isolated from a cDNA library of wild-type MEFs by polymerase chain reaction (PCR) using Phusion (FINNZYMES). CNOT3 mutants were generated by PCR using full length CNOT3 cDNA as a template. cDNAs were inserted into pMXs vectors (Morita et al., 2007).

Binding analysis between 3'UTRs of mRNAs and protein

3'UTRs of mRNAs were isolated from total RNA MEF by PCR. The length of 3' UTRs is as follows. *creb3*: 266 bases, *pik3c3*: 386 bases, *ripk1*: 2,390 bases (RIPK1-1, RIPK1-2, RIPK1-3, RIPK1-4 and RIPK1-5 correspond to 1-520, 473-990, 940-1,460, 1,410-1,930 and 1,880-2,390, respectively). FLAG peptide-conjugated RNA was generated as previously described (Adachi et al., 2014). MEFs were lysed with lysis buffer (1% NP-40, 50 mM Tris–HCl [pH 7.5], 150 mM NaCl, 1mM EDTA, 1 mM phenylmethylsulfonylfluoride, 10 mM NaF). Lysates were incubated with FLAG-tagged RNA for 2 h. Binding proteins were purified with anti-FLAG antibody (M2)-conjugated agarose and analyzed by immunoblot.

Gel filtration chromatography

MEFs were lysed with buffer (0.5% NP-40, 50 mM Tris–HCl [pH 7.5], 150 mM NaCl,

1 mM EDTA, 10 mM NaF). Lysates (0.5 mL, 0.4 mg/mL) were applied to a Superose™ 6 10/300 GL column using an AKTA Pure (GE healthcare). The flow rate was 0.5 mL/min and 0.5 mL fractions were collected.

Quantitative PCR primers

gene	forward	reverse
gapdh	ctgcaccaccaactgcttag	gtcttctgggtggcagtgat
cnot1	agaacctggctgtggaccta	tgagtgtggctgtttgggta
cnot2	cagacccaggaatggtacatc	ggtgatgcaaatttgggatag
cnot3	aagaagaaaggcgacaagga	gtccagcattcgcaggat
cnot6l	cctcgcagaatttacaccatc	ttaagctccgcactctaccc
cnot7	ccaggcaggatctgactcac	tgaccacagtatttggcatca
cnot8	gcaggctcagactctctgct	tagaggcgcccacaatactt
cnot9	gtctgcgcatcatggagtc	aaccagtgtcatccaagagga
cnot10	acaaggcccgaaagtgtct	aaggtagacagccagcagga
dvl2	acttcaccctccctcgaaa	gaggagccagggtaagcag
sirt5	ccagctttagcaggaaaagg	gactgggattctggcgtct
cdkn1a	ttgccagcagaataaaaggtg	tttgctcctgtgcggaac
ripk3	aggcttctaaagcgagtgatgt	tgaagtcttgtctaccaactcagc
ripk1	tacctccgagcaggtcaaat	aaaccaggactcctccacag
pik3c3	agagcgtccacgcactgt	tcccctttcattttctccagt
creb3	ccctcaacccttcctctca	cttgcggctttcttgagc
zfp292	cctggttttgtgtactgccttt	tgctgcaatttactccaaaaca
klf9	ctcagaactgcttttaacattaggg	aacactttcctttttagctcgtg
fbxo30	gagaagccagggtttgagc	tcatacagtgtgagtgctgctg

Primers for poly(A) tail length assay

gene	forward	reverse
ripk1	gtatgagttgaaggatggtcagta	tagtcacataaattttattcagtactc
ripk3	agagaatcactgcaagagcct	cacagttaacatgctatgtttattga
pik3c3	ctgctgtgtactaaagacatcaaag	tcagagggtaaaatgctttactgtttc

Primers for RNA-IP-PCR

gene	forward	reverse
ripk1	cacggaattcgtatgagttgaaggatggtc	gagtctcgagcagtactctgtatttaaagt
ripk3	gctcgaattcacttcagagaatcactgcaa	cacagttaacatgctatgtttattga
pik3c3	cagtgaattcaaggggatttgacccatcct	atcgcggccgctcagagggtaaaatgctt
sirt5	cagcgaattccatgatggatgtgtattatt	atgctcgagacctaccggggaggtttgtc
creb3	caacgaattcgtgtgaggatgtgggtgtc	gactgcggccgcgaggaaccacttatttat
dvl2	gtaggaattcggacccatcttgaggccaca	caatgcggccgcggcctctatttgctttat
cdkn1a	gcacgaattcgctattcctgattgttttct	gatcgcggccgcttttaagtacccacgagc
gapdh	gtacgaattcccggggctggcattgctctc	gctactcgaggatggtattcaagagagtag

Supplementary Figure legends

Supplementary Figure 1. Decrease of CCR4-NOT complex subunits upon CNOT3 depletion is not recovered by a proteasome inhibitor.

CNOT3$^{loxP/loxP}$ MEFs were infected with mock or Cre-expressing retrovirus. Cell lysates were prepared after treatment with DMSO or MG132 (50 μM) for 3 h, and were analyzed by immunoblot.

Supplementary Figure 2. Caspases are not cleaved in CNOT3-depleted MEFs.

Lysates from CNOT3$^{loxP/loxP}$ MEFs infected with mock (control) or Cre-expressing retrovirus (CNOT3KD) were analyzed by immunoblot.

Supplementary Figure 3. Cells that survive even after cell death stimulation

express CNOT3.

MEFs infected with control (Mock) or Cre-expressing retrovirus (Cre) were treated with zVAD (+: 40 μM). DMSO was used as a control treatment (-). Three days later, surviving cells were collected and lysed. Cell lysates were analyzed by immunoblot. The results from two independent experiments are shown. Note that CNOT3 level was low in the absence of zVAD because significant populations of CNOT3KD MEFs were alive owing to caspase-mediated, anti-necroptotic pathway.

Supplementary Figure 4. Increased candidate CCR4-NOT targets in CNOT3-depleted MEFs.

(A) qPCR analysis of mRNAs in control or CNOT3-depleted MEFs (CNOT3KD). *gapdh* mRNA levels were used for normalization. n=3 for each genotype. All values represent the mean+sem. $^{**}P<0.01$; $^{***}P<0.001$

(B) Lysates from $CNOT3^{loxP/loxP}$ MEFs infected with mock (control) or Cre-expressing retrovirus (CNOT3KD) were analyzed by immunoblot.

Supplementary Figure 5. Longer half-lives of CCR4-NOT target mRNAs in CNOT3-depleted MEFs. CNOT3 binds to the targets.

(A) Control and CNOT3KD MEFs were treated with Act. D. Relative mRNA levels were determined by qPCR at 4 h time intervals after Act. D treatment and normalized to the *gapdh* mRNA level. mRNA level without Act. D treatment (0h) was set to 100%. n=3 for each genotype. All values represent means ±sem. **P*<0.05; ***P*<0.01; ****P*<0.001

(B) Lysates from MEFs were immunoprecipitated with control Ig or anti-CNOT3 antibodies. Immunoprecipitates were analyzed by RT-PCR using primers for 3'UTRs in *creb3*, *dvl2*, *sirt5*, and *cdkn1a* mRNAs.

Supplementary Figure 6. Ago2 or an AU-rich element binding protein ZFP36L1 bind to the 3'UTR of CCR4-NOT target mRNAs.

(A,B) FLAG peptide covalently linked to the 3'UTRs of mRNAs was incubated with lysates from MEFs. Because the 3'UTR of *ripk1* mRNA is very long, it was divided into five fragments (RIPK1-1 ~ RIPK1-5) and used for the analysis (A). Bound proteins were purified using anti-FLAG antibody-conjugated agarose and analyzed by immunoblot.

Supplementary Figure 7. Excess CNOT3 forms an oligomer in addition to the

CCR4-NOT complex.

(A) Lysates from MEFs infected with mock or WT CNOT3WT-expressing retrovirus were fractionated by gel filtration chromatography and analyzed by immunoblot.

(B) Fractions indicated in (A) (* or **) were immunoprecipitated with anti-Flag antibody. Immunoprecipitates were loaded onto SDS-polyacrylamide gels and silver stained. Note that CNOT1 and CNOT3 exist at a stoichiometry of 1:1 in the immunoprecipitates from * fraction, while the immunoprecipitates from ** fraction consist almost entirely of CNOT3.

Supplementary Figure 8. RIPK3 suppression restores viability of CNOT3KD MEFs.

Growth curves corresponding to CNOT3$^{loxP/loxP}$ MEFs transduced with retroviruses (shRNA constructs) and adenoviruses (LacZ or Cre) in the presence of DMSO or zVAD treatment. Each time point was determined in triplicate. All values represent means ±sem. ***P*<0.01; ****P*<0.001

Supplementary Figure 9. Nec-1 does not suppress necroptosis of CNOT3KD MEFs in the absence of RIPK1.

(A) Morphology of CNOT3KD MEFs infected with retrovirus-expressing shControl or shRIPK1, treated with zVAD alone or zVAD and Nec-1 for 24 h.

(B) Cell death assessed by PI uptake via flow cytometry of CNOT3KDMEFs transduced with retrovirus expressing shRNA constructs in the presence of zVAD alone or zVAD and Nec1 for 24 h. n=3. All values represent means ±sem. ***P<0.001

Supplementary Table legends

Supplementary Table 1. Genes stabilized in CNOT3-depleted MEFs.

Lists of genes stabilized in CNOT3-depleted MEFs, determined by microarray analysis. Normalized expression levels in control and CNOT3-depleted MEFs, treated with actinomycin D, are shown. See also Figure 4

Supplementary Table 2. Genes stabilized and increased in CNOT3-depleted MEFs.

Gene lists, magnitude of changes (CNOT3-depleted MEFs/control MEFs), and normalized expression values are shown. Genes that increased more than 1.5-fold in CNOT3-depleted MEFs compared to control MEFs are shown in Figure 4.

Supplementary Table 3. Functional annotation of genes stabilized in CNOT3KD MEFs.

Genes selected in Figure 4 were subjected to Ingenuity Pathway Analysis to identify specific functions. Functions, p-values, the number of genes, and gene lists categorized in each function are shown.

Supplementary Figure 1

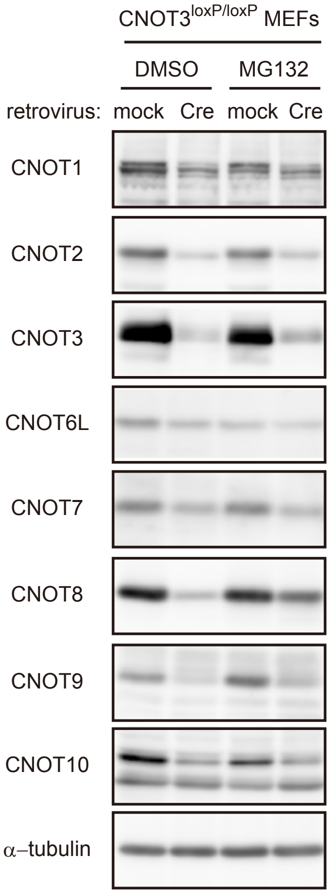

Supplementary Figure 2

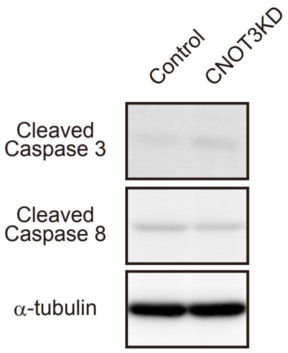

Supplementary Figure 3

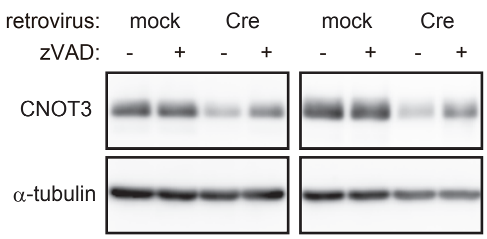

Supplementary Figure 4

A

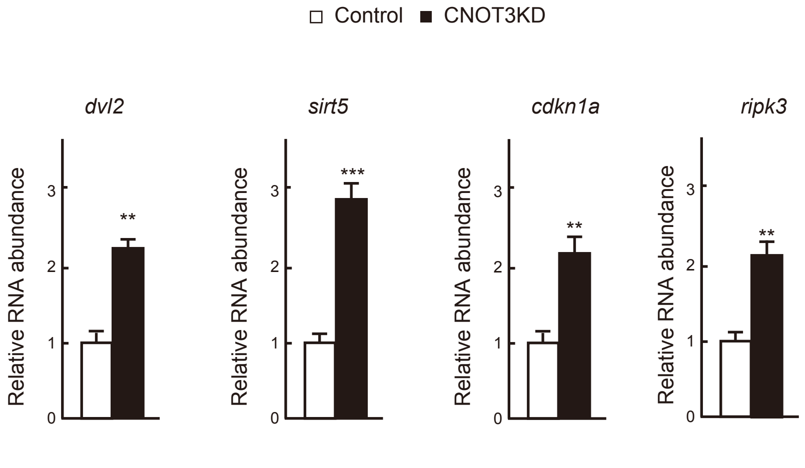

B

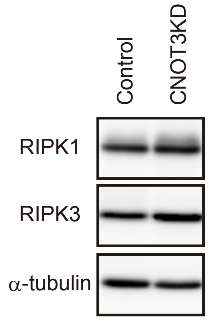

Supplementary Figure 5

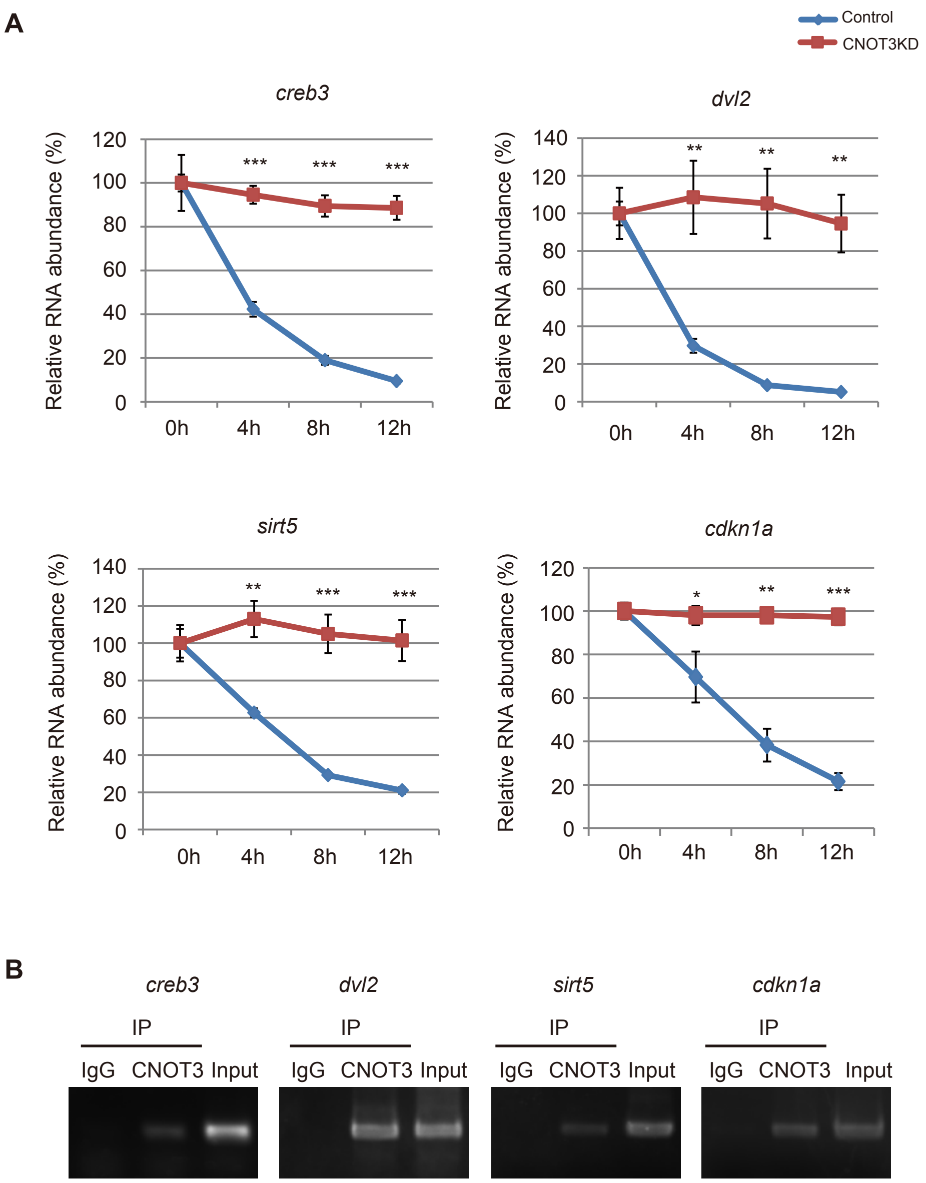

Supplementary Figure 6

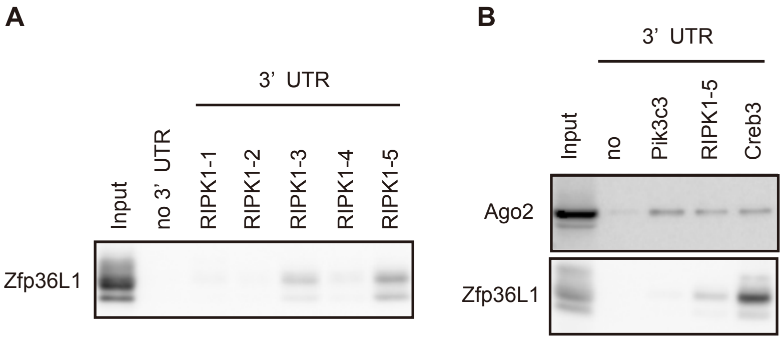

Supplementary Figure 7

A

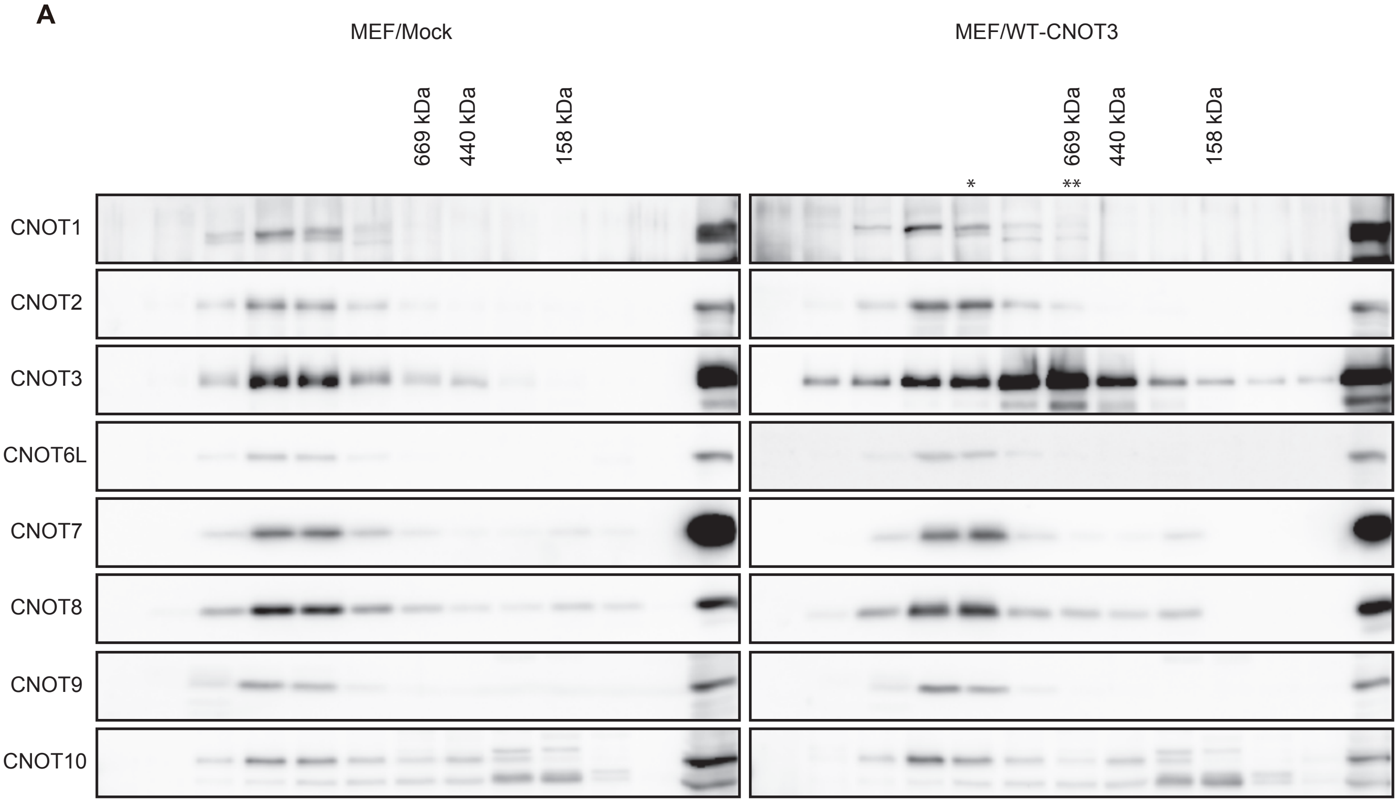

B

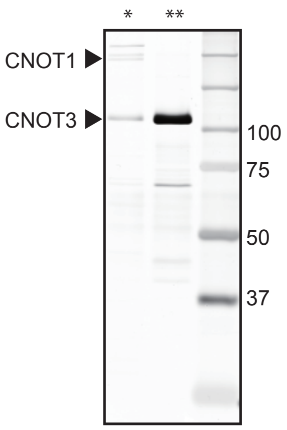

Supplementary Figure 8

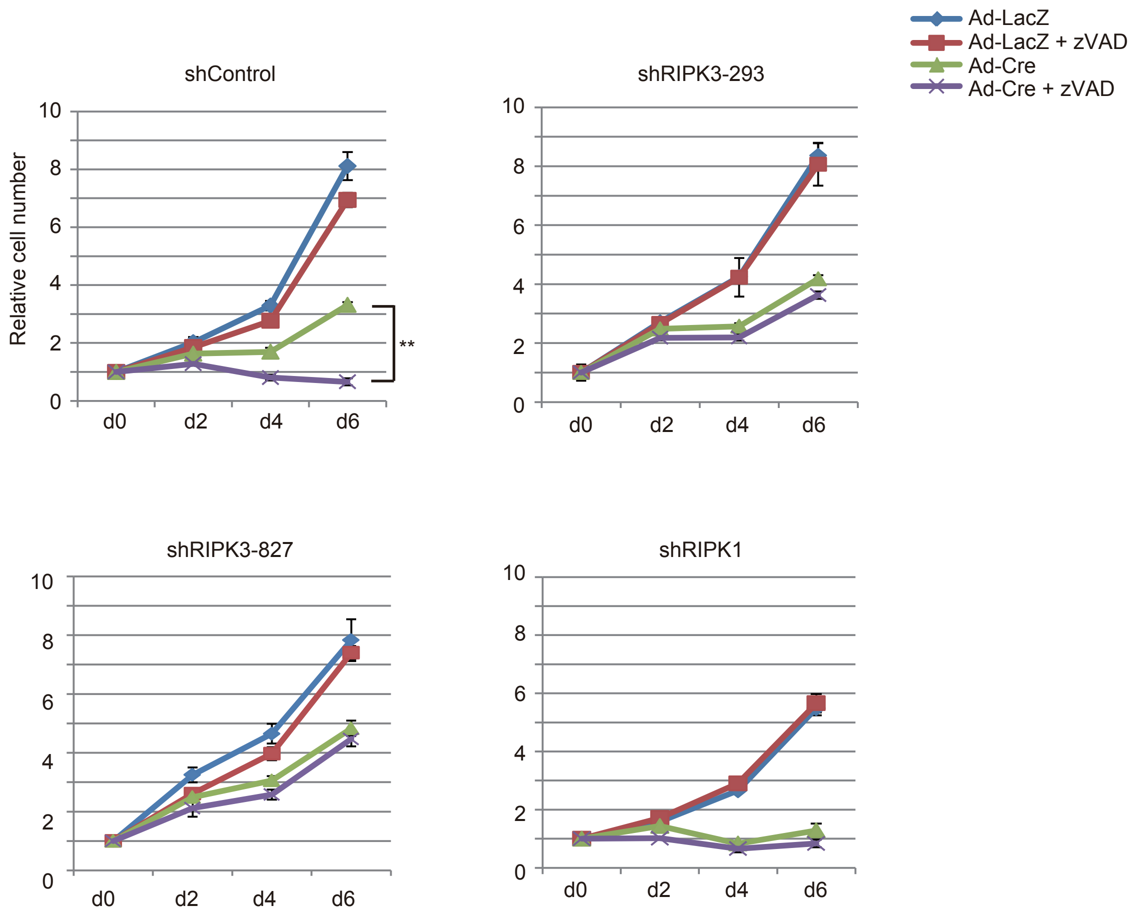

Supplementary Figure 9

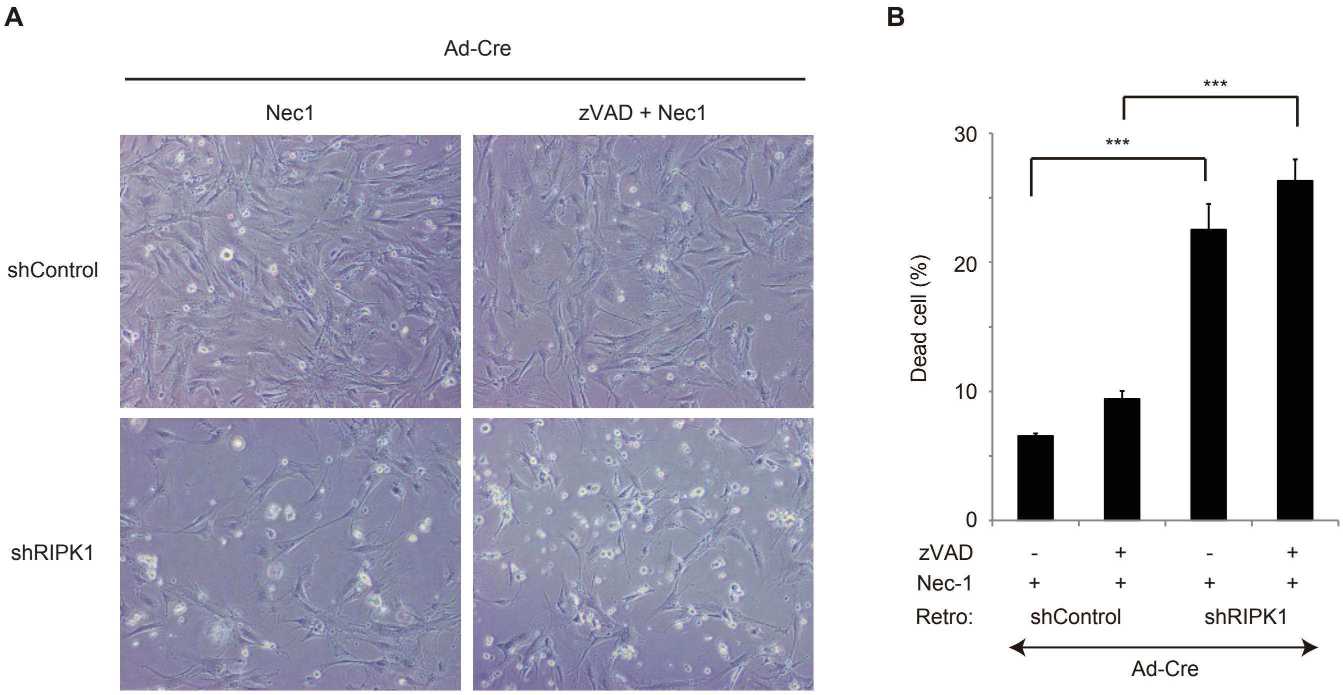

Supplement: Supplementary Information [file srep14779-s1.pdf]
